# Advances in materials science for ocular diseases induced by cardiovascular risk factors

**DOI:** 10.3389/fbioe.2025.1618232

**Published:** 2025-06-27

**Authors:** Peiwen Chen, Bo Zheng, Peng Wang, Hao Liu, Haifeng Pei

**Affiliations:** ^1^ School of Medicine, University of Electronic Science and Technology of China, Chengdu, China; ^2^ Department of Neurology, Zigong Third People’s Hospital, Zigong, China; ^3^ Department of Cardiology, The General Hospital of Western Theater Command, Chengdu, China; ^4^ College of Medicine, Southwest Jiaotong University, Chengdu, Sichuan, China

**Keywords:** cardiovascular risk factors, ocular diseases, materials science, smart sensors, drug delivery systems, tissue repair

## Abstract

Cardiovascular risk factors such as hypertension, hyperlipidemia, and hyperglycemia are closely associated with ocular diseases including glaucoma, diabetic retinopathy, and dry eye syndrome. These conditions are characterized by microvascular damage, hemodynamic alterations, and pathological neovascularization, ultimately leading to significant visual impairment. Traditional treatments often suffer from limitations, such as invasiveness and poor target specificity, highlighting the urgent need for innovative therapeutic approaches. Recent advancements in biomaterials have substantially improved therapeutic efficacy, particularly in the areas of targeted drug delivery, smart sensors, and tissue repair. Smart sensors like contact lenses enable continuous monitoring of intraocular pressure, enhancing glaucoma management. Nanotechnology and drug delivery systems improve drug targeting and bioavailability, enhancing anti-angiogenic therapies. Additionally, biocompatible materials and nanomaterials have shown promise in promoting retinal and optic nerve repair, facilitating neural regeneration and reducing aberrant neovascularization. Despite ongoing challenges, the rapid evolution of materials science holds transformative potential for developing more effective and personalized treatments for ocular diseases.

## 1 Introduction

Ocular diseases increasingly reflect the systemic burden of cardiovascular risk factors - including hypertension, hyperlipidemia, hyperglycemia, and hyperuricemia (Modjtahedi et al., 2016; Skrzypecki et al., 2019; Wang and Bao, 2019; Liu L. et al., 2020; Zhou et al., 2022)—which contribute to visual impairment through mechanisms such as microvascular dysfunction, chronic inflammation, and metabolic stress. These systemic conditions result in retinal damage via pathways involving oxidative stress and microvascular injury. For example, hypertension induces abnormal shear stress in retinal vessels, leading to endothelial dysfunction and neovascularization, while hyperglycemia disrupts the blood-retinal barrier through VEGF imbalance and inflammatory pathways (Liu L. et al., 2020; Jiang et al., 2024). Hyperlipidemia promotes lipid deposition, oxidative stress, and dysfunction of corneal endothelial pumps, ultimately reducing cell density, impairing intercellular junctions, and diminishing regenerative capacity—highlighting for the first time that the corneal endothelium is a previously unrecognized target tissue of hyperlipidemic injury (Bu et al., 2020). As the global prevalence of chronic systemic diseases continues to rise, ocular complications are becoming increasingly common, often coexisting in patients with complex cardiometabolic profiles.

Cardiovascular risk factors exert widespread effects on both the anterior and posterior ocular segments. These factors not only contribute to microvascular dysfunction in the retina but also impair anterior structures such as the corneal endothelium and uveal blood flow. Corneal endothelial dysfunction reduces transparency and disrupts fluid regulation, while uveal hypoperfusion compromises nutrient delivery to intraocular tissues (Aşıkgarip et al., 2022; Zeng et al., 2022). Importantly, systemic vascular conditions such as hypertension are strongly associated with retinal vascular occlusions, underscoring a unified pathophysiological mechanism in which cardiovascular dysregulation leads to vascular insufficiency and ischemic damage across the entire ocular system (Cheung et al., 2012).

Traditional ocular therapies often limited by factors such as invasive delivery, low bioavailability, and poor patient compliance. Standard treatments—including anti-VEGF agents and laser photocoagulation—face therapeutic ceilings and procedural burdens that restrict their long-term efficacy. In this context, materials science offers powerful interdisciplinary solutions by enabling precise drug delivery, real-time biosensing, and bioengineered tissue regeneration. Recent breakthroughs include microenvironment-responsive smart materials, on-demand drug release systems, and integrated physiological monitoring technologies. Smart contact lenses and injectable hydrogels exemplify these advances, providing improved drug retention, enhanced tissue integration, and personalized treatment modalities (Zhu et al., 2022; Wang L. et al., 2024). Emerging technologies such as smart contact lenses, sustained-release platform, and nanostructured hydrogels are expanding therapeutic possibilities for ocular diseases, particularly those linked to systemic dysfunction (Liu J. et al., 2020; Jiang et al., 2024).

**FIGURE 1:**
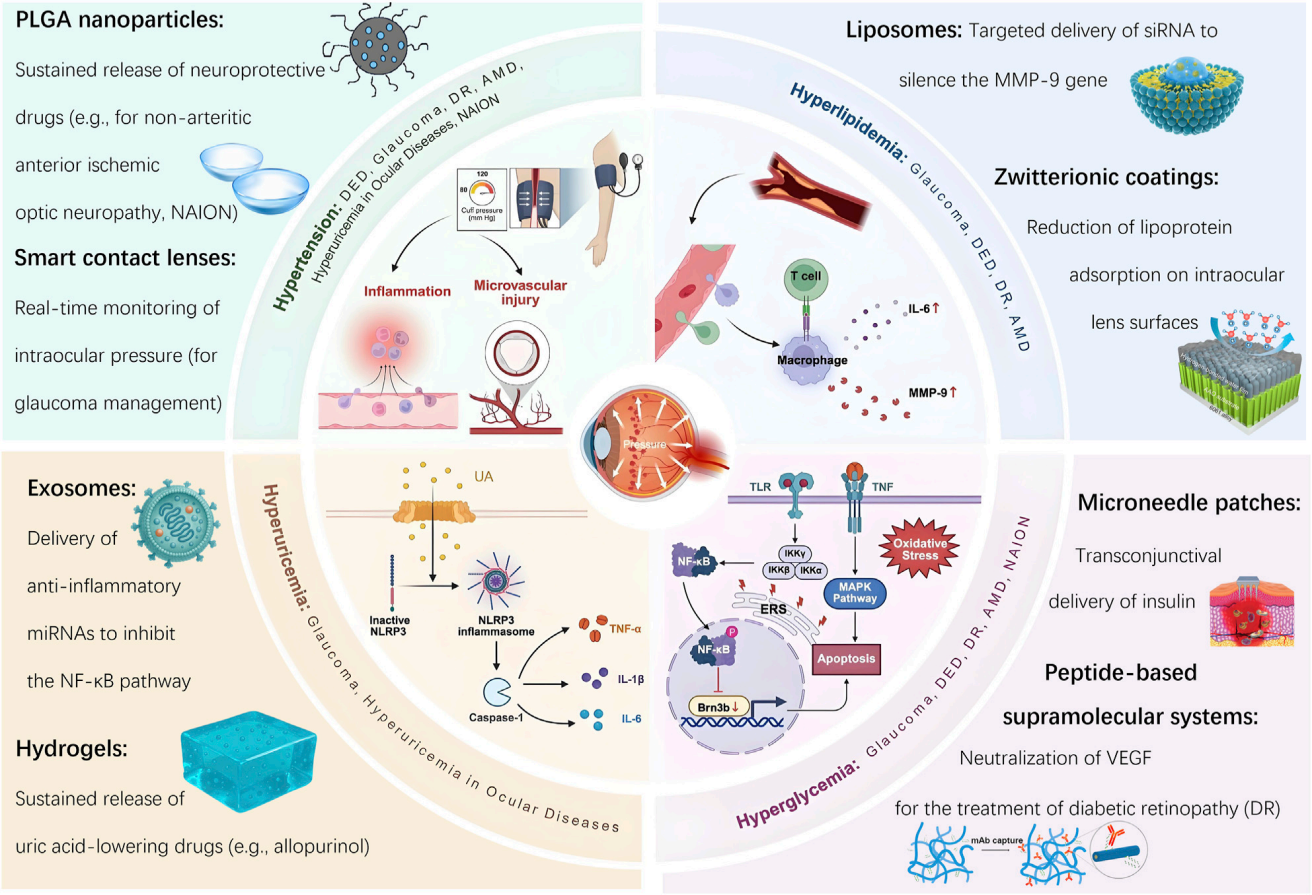
Bioaterial-based interventions for ocular diseases induced by cardiovascular risk factors.

This review presents a comprehensive analysis of six major ocular diseases from a dual perspective: the pathogenesis driven by cardiovascular risk factors and the therapeutic opportunities enabled by advanced biomaterials. For each condition, we discuss the underlying mechanisms, current treatment challenges, and how materials science offers targeted, functional, and clinically relevant interventions. Finally, we highlight existing challenges and propose future directions for translational application and interdisciplinary research (Figure 1).

## 2 Dry eye disease

The pathogenesis of Dry Eye Disease (DED) is influenced by systemic conditions such as hypertension, hyperlipidemia, and hyperglycemia, which exacerbate disease progression through mechanisms involving chronic inflammation and oxidative stress (Thang et al., 2024; Tran Tat et al., 2024; Park and Park, 2016; Su et al., 2022). Recent advances in materials science—particularly in targeted drug delivery systems and biosensing technologies—have introduced innovative diagnostic and therapeutic strategies that support the development of personalized treatment approaches.

### 2.1 Pathological mechanisms of dry eye disease induced by cardiovascular risk factors

Clinical evidence demonstrates a strong association between cardiovascular risk factors and the severity of DED. In patients with primary hypertension, the prevalence of DED reaches 41.7%, significantly higher than 18.8% observed in control groups (P < 0.001). Moreover, hypertension exhibits a stage-dependent relationship with DED prevalence, increasing from 27.1% in stage I to 57.6% in stage III hypertension (P < 0.001), with comorbid diabetes further elevating risk to 55.6% in T2DN patients (vs. 37.3% in general diabetics). Independent risk factors include advanced age, longer hypertension duration, concurrent diabetes, and elevated levels of plasma creatinine and high-sensitivity C-reactive protein (hs-CRP) (P < 0.001) (Thang et al., 2024; Tran Tat et al., 2024). Notably, antidiabetic medications choice may also influence DED risk—a recent study found that patients with type 2 diabetes initiating sodium-glucose cotransporter 2 inhibitors had a significantly lower incidence of DED compared to those receiving glucagon-like peptide-1 receptor agonists (9.0 vs. 11.5 cases per 1,000 person-years, HR = 0.78) (Su et al., 2022). The impact of cardiovascular conditions on DED is further exacerbated by comorbidities such as diabetes, which compromises ocular surface integrity through metabolic dysregulation. This indicates DED may serve as a manifestation of broader systemic dysfunction involving inflammation, vascular health and disturbances in the ocular microenvironment. Dyslipidemia also contributes significantly to the pathogenesis of DED and meibomian gland dysfunction (MGD). Elevated total cholesterol and triglycerides are associated with an increased risk of DED (OR = 1.6, 95% CI 1.2–2.1), with hypertriglyceridemia independently linked to DED symptoms in females (OR = 1.13) (Park and Park, 2016). While MGD is commonly related to lipid abnormalities, a cohort study suggests that dyslipidemia may be more closely tied to non-MGD forms of DED, possibly through mechanisms involving tear film instability or the upregulation of inflammatory markers such as interleukin-6 (IL-6) and matrix metalloproteinase-9 (MMP-9) (Mussi et al., 2021). This finding underscores the importance of a systems-based perspective on DED, recognizing the interconnection between cardiovascular risk factors and ocular surface health.

### 2.2 Materials science in dry eye disease

In response to the pathological mechanisms described above, materials science has introduced innovative strategies for the treatment of DED. These advancements have transformed DED management through two synergistic approaches: advanced diagnostic systems and targeted therapeutic platforms. Cutting-edge sensing technologies capable of multi-parameter detection and biomarker identification have greatly improved diagnostic accuracy. For example, smart contact lenses embeded with fluorescent corneal lenses enable quantitative analysis of tear film properties—such as pH and electrolyte concentration—through smartphone integration (Yetisen et al., 2020). This innovation is particularly relevant for patients with cardiovascular comorbidities, whose tear film dynamics and inflammatory responses may fluctuate due to systemic conditions like hypertension, diabetes, and dyslipidemia. Label-free biosensors based on localized surface plasmon resonance (LSPR), incorporating gold nanoshell-hydrogel composites (e.g., Al-OEGA-coated AuNSs, AuNS@PNM), detect tear proteins such as lysozyme and lactoferrin through covalent or electrostatic binding. These sensors generate linear LSPR wavelength shifts, enabling highly sensitive, portable detection of tear biomarkers. Such systems support early screening of chronic DED, facilitate grading of ocular surface inflammation, and promote personalized therapeutic strategies (Culver et al., 2018; Wechsler et al., 2021). Targeted drug delivery technologies address the limitations of conventional eye drops, which typically exhibit bioavailability below 5% and often lead to side effects. Advanced liposomal nanosystems target the ocular surface using electrostatic adhesion and lysosomal escape, co-delivering SS-31 peptides and insulin, demonstrating significant anti-inflammatory, antioxidant and mitochondrial repair effects that restore tear secretion, reduce pro-inflammatory cytokines (IL-1β, IL-6, TNF-α) and improve mitochondrial function in DED models (Xia et al., 2024). Contact lens-based sustained-release systems, utilizing silicone hydrogels or vitamin E-modified carriers, have been developed to extend the release duration of drugs such as cyclosporine A for up to 14 days. These platforms support long-term immunomodulation and ocular surface repair, while minimizing dosing frequency—an important factor in improving patient compliance in chronic disease management (Mondal et al., 2023). Furthermore, integrated theranostic systems that combine fluorescent sensing contact lenses with drug-loaded nanocarriers (e.g., lipid nanocapsules, micelles) offer real-time monitoring of tear film parameters alongside hydrophobic drug delivery. These systems extend ocular retention time and reduce administration frequency, enhancing both therapeutic efficacy and user convenience (Joshi et al., 2023). Despite these significant advancements, challenges related to sensitivity, comfort, and production cost continue to limit widespread clinical translation. Nevertheless, the integration of real-time diagnostics, targeted drug delivery, and sustained-release systems represents a promising paradigm shift in the management of ocular diseases influenced by cardiovascular risk factors.

## 3 Glaucoma

Glaucoma, a leading cause of irreversible blindness, is primarily associated with elevated intraocular pressure (IOP). Cardiovascular risk factors - such as hypertension, hyperlipidemia, and hyperuricemia - can impair ocular blood flow, contributing to abnormal increases in IOP and thereby elevating the risk of glaucoma development (Kuang et al., 2020; Wang L. et al., 2024; Zhou et al., 2022; Biggerstaff et al., 2021).

### 3.1 Pathological mechanisms of glaucoma induced by cardiovascular risk factors

Systemic hypertension exhibits a bidirectional relationship with IOP, with epidemiological studies demonstrating a 1.4-fold increased risk of primary open-angle glaucoma (POAG) (95% CI 1.2–1.7). This association is primarily mediated through impaired ocular hemodynamics and disrupted axonal transport in the optic nerve (Kuang et al., 2020; Wang L. et al., 2024). In addition to elevated blood pressure, dyslipidemia plays a significant role in the pathogenesis of glaucoma. Elevated triglyceride levels are associated with an increased risk of glaucoma (HR = 1.4, 95% CI: 1.2–1.7), and polymorphisms in the APOB gene further modulate susceptibility (Kang et al., 2024). Interestingly, statin use demonstrates population-specific effects, with increased glaucoma risk observed in individuals aged 60–69 or those with LDL-C levels ≥4.1 mmol/L (RR = 1.2, P = 0.04) (Lee et al., 2024). Disorders of glucose metabolism, particularly diabetes, also represent a major risk factor for glaucoma, though the underlying mechanisms are multifaceted. Diabetes significantly elevates glaucoma risk - as evidenced in the Blue Mountain Eye Study (OR = 2.12, 1997) - with hyperglycemia-induced retinal ganglion cell (RGC) apoptosis through endoplasmic reticulum stress (ERS). Notably, ERS inhibitors such as 4-phenylbutyric acid have been shown to reverse these effects (Zhou et al., 2022). Furthermore, hyperglycemia suppresses the expression of Brn3b via signaling pathways involving NO, NF-κB, and TNF-α, further accelerating RGC apoptosis (Tjandra et al., 2020). The risk is compounded by other metabolic syndrome components such as hypertension and prolonged hyperglycemia. Elevated fasting glucose levels and longer diabetes duration are strongly correlated with increased glaucoma risk (AlDarrab et al., 2023; Li et al., 2024). In postmenopausal women, the severity of diabetes is particularly impactful, with insulin use associated with a nearly twofold increased glaucoma risk (HR = 1.884) (Jung et al., 2021). Neovascular glaucoma, often secondary to diabetic retinopathy (DR) requires comprehensive management. However, genetic heterogeneity - such as GLIS3 mutations - and paradoxical findings, such as reduced open-angle glaucoma (OAG) risk in some diabetic populations, suggest that distinct molecular subtypes may underlie disease progression (Boddu et al., 2022; 2022; Virtanen et al., 2023). Overall, hyperglycemia promotes glaucoma development through mechanisms involving ERS, inflammation, and vascular injury, underscoring the importance of early screening and glycemic control (Yang et al., 2021). Among metabolic risk factors, uric acid (UA) plays a paradoxical and still controversial role. While gout has been associated with a 19% lower risk of POAG (HR = 0.81) (HR = 0.81) (Biggerstaff et al., 2021), lower serum UA levels have also been correlated with increased POAG risk (Serra et al., 2021). These findings suggest that uric acid may participate in glaucoma pathogenesis via complex inflammatory mediator networks, highlighting the potential role of anti-inflammatory strategies. Proinflammatory cytokines such as IL-6 and TNF-α serve as molecular bridges between cardiovascular and ocular diseases. IL-6 may enhance aqueous humor outflow in the short term but contributes to long-term trabecular meshwork damage, thereby impairing IOP regulation (Xiao et al., 2023). TNF-α facilitates RGC apoptosis and glaucomatous tissue injury by activating the NF-κB and MAPK signaling pathways (Li et al., 2021). Taken together, these findings reveal a complex, multifactorial interplay between cardiovascular risk factors and glaucoma development, mediated by cellular dysfunction, inflammatory responses, and vascular remodeling. Understanding these mechanisms underscores the importance of exploring molecular pathways and investigating innovative material-based strategies targeting these pathological processes. Ultimately, an integrated, systemic-ocular approach is essential to improve clinical outcomes in glaucoma management.

### 3.2 Application of materials science in glaucoma

Given the influence of cardiovascular comorbidities such as hypertension and diabetes on the pathogenesis of glaucoma, biosensing technologies that integrate intraocular pressure (IOP) monitoring with metabolic parameters (e.g., tear glucose and inflammatory cytokines) represent promising tools for early detection and intervention in patients at cardiometabolic risk. Sensing technologies have rapidly advanced in medical applications, especially for real-time disease monitoring and therapeutic guidance. Modern systems now combine biosensors with data acquisition platforms, enabling continuous, real-time tracking of IOP and other relevant parameters (Shean et al., 2024; Shao et al., 2025). Compared to traditional diagnostic approaches, these technologies support more precise, personalized, and multi-parametric health management (Shin et al., 2021). As mechanistic understanding of cardiovascular–IOP interactions deepens, intelligent sensors are becoming indispensable in translating this knowledge into individualized glaucoma care. Nonetheless, limitations remain, including suboptimal sensitivity, limited stability, constrained integration capacity, and dependence on single time-point measurements—as typified by Goldmann applanation tonometry (Moses, 1958). To address these challenges, smart contact lenses incorporating flexible sensors and wireless communication modules have been developed (Kim et al., 2022; Zhu et al., 2022). Innovative sensor designs using silver nanowires and hollow gold nanowires have demonstrated excellent sensitivity and biocompatibility for ocular applications (Kim et al., 2021; Kim et al., 2022). Most significantly, the hollow gold nanowire-based design achieves 11%–25% greater sensitivity compared to conventional thick Parylene C substrates when measuring equivalent IOP levels. Now providing 24-h IOP monitoring, facilitating early glaucoma detection and personalized treatment (Zhang J. et al., 2022). These platforms are evolving into multifunctional systems, where sensor feedback can trigger on-demand drug release (Kim et al., 2022), Hydrogel-based biosensors—such as those using Ti_3_C_2_Tx MXene—allow for simultaneous detection of tear glucose and IOP, supporting remote monitoring and real-time health display (Kim et al., 2017; Zhu et al., 2022; Duan et al., 2024). These multifunctional platforms enable long-term, non-invasive, real-time continuous IOP monitoring, marking a transformative shift in glaucoma diagnosis and the broader management of chronic diseases.

Drug delivery technologies are also crucial in addressing the limitations of conventional treatments, particularly for glaucoma and diabetic retinopathy. Ocular anatomical barriers—such as tear turnover and the blood–retinal barrier—severely restrict the bioavailability of topical agents, often reducing it to below 5% (Tomi and Hosoya, 2010). Therefore, innovative delivery platforms are essential for enhancing drug retention, decreasing dosing frequency, and minimizing systemic exposure (Akulo et al., 2022; Lin et al., 2023). Cardiovascular risk factors exacerbate glaucoma progression through multiple pathways (see Section 2.1), making precisely targeted delivery systems imperative: Nanocarrier platforms utilizing extracellular vesicles and exosomes can encapsulate RNA, proteins, and lipids, thereby enhancing drug targeting while minimizing side effects (Liu J. et al., 2020; Durmaz et al., 2024). Furthermore, Brugenera et al. developed a novel preservative-free liposomal delivery system (LAT-HA-LIP) that simultaneously achieves sustained IOP control and protects the ocular surface—addressing the limitations of conventional anti-glaucoma eyedrops, which may destabilize the tear film and cause DED due to preservative toxicity (Brugnera et al., 2025). Moreover, recent studies show that core-shell nanoparticles with PLGA carriers achieve 1.8-fold higher bioavailability in the choroid compared to PLA carriers when delivered via the conjunctival-scleral pathway (p = 0.003), offering new therapeutic strategies for posterior segment diseases (Mahaling and Katti, 2016). This underscores a shift toward more precise, controlled, and sustainable drug delivery, with nanotechnology enhancing both drug penetration and the safety profile of ocular therapies. Periocular routes—such as subconjunctival injections—also enable sustained drug delivery to the posterior segment, offering a minimally invasive yet effective approach (Rafiei et al., 2020). Sustained-release technologies, including drug-loaded contact lenses, wearable devices, and intraocular implants, offer precise control over drug release kinetics and improve patient adherence (Kompella et al., 2021; Al-Qaysi et al., 2023). Hydrogel-based systems and dendritic polymers—owing to their high water content, drug-protective capacity, tunable release profiles, and anti-inflammatory properties—optimize retention on the ocular surface (Akulo et al., 2022; Wang et al., 2023). Overall, these systems allow efficient penetration into ocular tissues and represent a significant improvement over traditional topical therapies.

Tissue repair materials are widely applied in ophthalmology, especially for managing postoperative scarring and neurodegeneration in glaucoma. Chronic inflammation induced by cardiovascular risk factors exacerbates fibrosis following glaucoma surgery. These biomaterials work by accelerating tissue healing, modulating inflammation, and guiding cell growth using physical scaffolds or bioactive agents. However, challenges remain regarding biodegradability, long-term efficacy, and target specificity. Advances in biomaterials, nanotechnology, and bioengineering have greatly expanded their clinical potential (Kompella et al., 2021; Sun et al., 2021). In glaucoma surgery, photo crosslinkable hydrogels such as GelDex-S58 inhibit postoperative fibrosis by controlling TGF-β signaling (Lin et al., 2023). Likewise, RGD (arginine–glycine–aspartate)-functionalized hydrogels target β1-integrin/FAK/Akt signaling pathways to suppress Tenon’s fibroblast activation, thereby reducing fibrotic scar formation (Chen B. et al., 2024). Beyond surgical applications, hydrogels can serve as reservoirs for sustained drug delivery. Mitomycin C (MMC)-loaded hydrogels, including those combined with RGD-modified carriers, provide prolonged anti-fibrotic effects with fewer side effects compared to conventional MMC treatments (Tu et al., 2023; Wu et al., 2023). Importantly, hypertension—a key cardiovascular risk factor—can directly elevate IOP through mechanical compression of the optic nerve or induce ischemic optic nerve damage via microvascular pathologies. Thus, in addition to structural repair, thermosensitive hydrogels loaded with neuroprotective agents have been developed. These hydrogels not only promote optic nerve regeneration but also help preserve visual function (Wang L. et al., 2024). With sensor integration, these materials provide a platform for real-time, feedback-controlled therapy—supporting both tissue recovery and functional vision preservation.

## 4 Ocular diseases related to hyperuricemia

Hyperuricemia, defined by elevated serum uric acid (UA) levels, has been implicated in a variety of systemic conditions, including gout, renal dysfunction, and cardiovascular diseases. Emerging evidence suggests that hyperuricemia is also linked to several ocular disorders, such as age-related macular degeneration (AMD), diabetic retinopathy (DR), and glaucoma. Elevated UA levels may contribute to the development and progression of these ocular pathologies by inducing oxidative stress, promoting inflammatory responses, and compromising the integrity of the blood-retinal barrier (Biggerstaff et al., 2021; Serra et al., 2021).

### 4.1 Pathological mechanisms of hyperuricemia in ocular diseases

Elevated serum uric acid (UA) contributes to ocular damage through multiple pathogenic pathways. High UA levels can promote endothelial cell dysfunction and increase vascular permeability, thereby accelerating the progression of DR and other retinal disorders (Ao et al., 2017; Li et al., 2019). Hyperuricemia exacerbates retinal microvascular and neuronal damage, with distinct mechanistic insights emerging from recent studies. Lu et al. (2022) reported an 18.3% reduction in superficial capillary plexus density among hyperuricemic women (p < 0.01), showing a significant linear correlation with serum urate levels (β = −0.24, p = 0.003). Complementarily, Yang et al. (2023) found that in males, each 1 mg/dL increase in serum UA was associated with a 13% increase in deep retinal capillary non-perfusion areas (OR = 1.13, 95% CI 1.05–1.22). Wei et al. (2023) further demonstrated that hypertensive patients with cerebral white matter lesions exhibited impaired macular microvascular architecture (FD-300, r = −0.41, p = 0.007), implicating disruption of the blood–retinal barrier.

Gout, a condition intrinsically linked to hyperuricemia, also presents with distinct ocular manifestations. Studies by Sharon and Schlesinger and Ao et al. (Sharon and Schlesinger, 2016; Ao et al., 2017) reported conjunctival urate crystal deposits in 33% of patients and neurotrophic dry eye in 22%. Karti et al. (2024) quantified retinal neurodegeneration in gout patients, showing sectoral thinning of the retinal nerve fiber layer (RNFL: −9.6 μm nasal) and ganglion cell complex (GCC: −12.3 µm inferior; p ≤ 0.005). These findings suggest that crystalline deposition within ocular tissues can provoke both inflammation and neurodegeneration. Meyer et al. (2024) provided pathological evidence of this process through a case of eyelid tophus, linking IL-1β–driven M1 macrophage polarization to chronic ocular inflammation. UA crystals activate the NLRP3 inflammasome, leading to the release of pro-inflammatory cytokines such as IL-1β, IL-6, and TNF-α. These mediators contribute to retinal neovascularization and macular edema in conditions such as DR and AMD (Thounaojam et al., 2019). In the pathogenesis of OAG, UA induces oxidative stress in the trabecular meshwork, impairing aqueous humor outflow and leading to elevated IOP. Additionally, crystal deposition contributes to RGC apoptosis (Biggerstaff et al., 2021; Serra et al., 2021). Moreover, hyperuricemia may exacerbate AMD by promoting choroidal inflammation. Elevated UA levels trigger the release of inflammatory mediators in the retina, damaging retinal pigment epithelial (RPE) cells and fostering the development of choroidal neovascularization (Pai et al., 2021; Pai et al., 2024). These findings underscore the dual role of UA in AMD - as both a systemic marker of oxidative stress and a direct mediator of ocular tissue injury - contributing to disease mechanisms across both anterior and posterior segment disorders.

### 4.2 Materials science in the treatment of hyperuricemia-related ocular diseases

Emerging biomaterial-based strategies are transforming the management of hyperuricemia-related ocular pathologies through precision-targeted interventions. Hyperuricemia, which is closely linked to gout and ocular diseases such as uveitis and retinal vascular occlusion, can be effectively addressed using advanced drug delivery systems.

Hydrogel microneedles loaded with colchicine have shown promise in localized treatment by effectively reducing inflammatory cytokine levels (Jiang et al., 2023). Macrophage-targeted liposomes encapsulating melatonin have demonstrated the ability to reprogram macrophage metabolism, presenting a novel therapeutic approach for ocular inflammation (Ma et al., 2025). A chitosan-based microneedle platform co-delivering colchicine and uricase enables sustained drug release, thereby improving patient adherence and supporting systemic uric acid management (Yang et al., 2023). Manganese-doped albumin nanogels (MAGNs) loaded with berberine have also shown efficient bioavailability and targeted delivery to inflamed tissues, which could be beneficial in treating hyperuricemia-associated ocular inflammation (Sun et al., 2023). Furthermore, red blood cell-encapsulated uricase formulations extend circulation time and enhance uric acid reduction, offering a promising strategy for enzyme replacement therapy (Ban et al., 2024). Microneedles provide a controlled and localized drug delivery system, with potential applications for ocular diseases linked to hyperuricemia (Yi et al., 2024). Febuxostat-loaded microneedles and nanogels enhance drug bioavailability and penetration, offering a solution for treating hyperuricemia-related eye conditions (Patel and Thakkar, 2023; Khan et al., 2024). In conclusion, these advances in materials science offer effective, non-invasive treatment options for hyperuricemia-related ocular diseases by enhancing drug targeting, minimizing systemic side effects, and improving patient compliance.

## 5 Diabetic retinopathy

Diabetic retinopathy is the leading cause of vision loss among individuals with diabetes. As the global prevalence of diabetes continues to rise, the incidence of DR is also increasing. The hallmark pathological features of DR include microvascular damage and pathological neovascularization.

### 5.1 Pathological mechanisms of diabetic retinopathy induced by cardiovascular risk factors

The pathological mechanisms by which cardiovascular risk factors contribute to DR underscore the disease’s complexity and the profound influence of systemic health on ocular outcomes. Hypertension (HTN) independently elevates the risk of DR, with each 1-unit increase in systolic blood pressure variability associated with a 2% higher risk (RR = 1.02), and even high-normal blood pressure (≥120/80 mmHg) showing a significant association with DR incidence (aOR = 1.114) (Zhang et al., 2023; Noroozi et al., 2024). These findings suggest that effective DR management must incorporate systemic parameters such as blood pressure, as they play a critical role in modulating retinal damage progression. Mendelian randomization studies further support a causal relationship between HTN and DR, with elevated intraocular pressure (IOP) also contributing to DR risk (OR = 1.090) (Wang X.-F. et al., 2024). Hypertension accelerates both retinal neurodegeneration and microvascular damage, leading to decreased peripapillary retinal nerve fiber layer (pRNFL) thickness, ganglion cell complex thinning, and reduced microvascular density compared to normotensive individuals with DR. It also promotes disease progression from early arteriolar thickening to advanced capillary occlusion (Huang and Fawzi, 2024; Sung et al., 2024; Yu et al., 2025). While achieving blood pressure control (target <130/80 mmHg) reduces the risk of DR onset (RR = 0.78), its effect on disease progression is modest (RR = 0.94). Notably, up to 19.7% of diabetic patients with a disease duration of ≥8 years remain undiagnosed with HTN (Woodward et al., 2020; Qureshi et al., 2025), underscoring the need for integrated retinal and blood pressure monitoring. The progression from arteriolar thickening to capillary occlusion illustrates how vascular injury leads to impaired retinal perfusion, exacerbating ischemia and contributing to retinal degeneration.

Hyperglycemia also plays a central role in DR pathogenesis by amplifying oxidative stress and inflammation, largely via the *metabolic memory* effect (Taurone et al., 2020; Huang et al., 2024). Elevated glucose levels trigger pericyte apoptosis, blood-retinal barrier (BRB) disruption, and vascular leakage through several mechanisms: mitochondrial reactive oxygen species (ROS) generation, advanced glycation end-product (AGE)–receptor for AGE (RAGE) signaling, VEGF upregulation, and microglial exosomal release of miR-155 (Lin et al., 2021; Tang et al., 2023; Wang X. et al., 2024). These pathways synergistically exacerbate retinal ischemia, as evidenced by increased central foveal thickness (∆ = +45 μm, P = 0.002) (Wang X. et al., 2024). Thus, comprehensive DR management must address not only local retinal pathology but also systemic metabolic and hemodynamic dysfunction.

Among ocular diseases associated with cardiovascular risk factors, DR is one of the most prevalent and severe. Sensing technologies are proving highly valuable in the monitoring, diagnosis, and treatment of such conditions (Keum et al., 2020). While current treatments primarily rely on intravitreal injection of anti-VEGF antibodies, long-term use of these agents can result in complications such as endogenous endophthalmitis (Li Y.-N. et al., 2023). Consequently, there is an urgent need for non-invasive diagnostic and therapeutic alternatives. To overcome the limitations of conventional injection-based therapies, nanoparticle drug delivery systems offer unique advantages, including targeted delivery and sustained release. For example, core-shell polycaprolactone/Pluronic^®^ F68 nanoparticles loaded with triamcinolone acetonide alleviate both inflammation and vascular abnormalities (Mahaling et al., 2018) loaded with triamcinolone acetonide simultaneously alleviate inflammation and vascular abnormalities. IL-12-loaded polymeric nanoparticles (IL-12-PNPs) inhibit VEGF-A and MMP-9 expression, helping to restore retinal thickness (Zeng et al., 2019). Fenofibrate-loaded nanoparticles (Feno-NPs) maintain therapeutic efficacy for up to 60 days after a single injection, reducing vascular leakage and neovascularization (Qiu et al., 2019). Chitosan/PLGA-based hydrogels delivered via subconjunctival injection modulate the VEGF/Occludin balance and reduce retinal apoptosis (Rong et al., 2019). Magnetic nanoparticle-optical coherence tomography (OCT) conjugates improve drug activity by over 100-fold and achieve targeted distribution to the retina (Amato et al., 2020). These advanced delivery systems integrate anti-inflammatory, anti-angiogenic, and neuroprotective mechanisms, offering sustained drug release (ranging from weeks to months) with minimal invasiveness via intravitreal or subconjunctival routes. As a result, they significantly enhance bioavailability and reduce treatment-associated risks, pushing DR therapy toward a precision medicine model. Emerging solutions include quantum dot-based immunosensors for tear biomarker detection in diabetic retinopathy (LOD = 110 pg/mL) and SGLT2 inhibitors reduce 46% ROS accumulation and significantly attenuate retinal apoptosis independent of glucose lowering, through the ERK1/2–cPLA2–AA–ROS signaling cascade (Wang et al., 2017; Hu et al., 2022).

In parallel, non-invasive treatment strategies have made notable progress. Smart supramolecular peptide-based eye drops capable of selectively binding soluble Semaphorin 4D effectively reduce pathological retinal neovascularization and leakage in DR models (Li Y.-N. et al., 2023). These innovations complement nanoparticle-based therapies, with sensing technologies - such as near-infrared contact lenses and fingertip AGE detectors-enabling early-stage diagnosis, while drug delivery systems target mid- and late-stage pathology through multi-target interventions (anti-inflammatory, anti-angiogenic, neuroprotective). The integration of sensor arrays with machine learning has further enabled rapid, cost-effective, and reliable diagnostic tools for diabetes and DR, particularly via non-invasive biomarker detection (Faura et al., 2022). These material-science - driven advancements not only improve patient compliance but also establish a new paradigm for DR management by precisely modulating key pathological pathways, including VEGF, ICAM-1, and Occludin.

## 6 Age-related macular degeneration

Age-related macular degeneration is the leading cause of vision loss in the elderly population. In addition to increasing the risk of cardiovascular diseases, cardiovascular risk factors are closely linked to both the onset and progression of AMD (Chen et al., 2023; Nahavandipour et al., 2020; Yadav et al., 2024). The underlying mechanisms connecting these conditions involve metabolic dysregulation, chronic inflammation, and vascular damage, which interact in complex and synergistic ways.

### 6.1 Pathological mechanisms of AMD induced by cardiovascular risk factors

Hypertension significantly increases the risk of wet age-related macular degeneration (wAMD). It is also associated with a greater need for anti-VEGF treatments, largely due to choroidal endothelial dysfunction. The impact of pharmacological interventions varies: β-blockers such as propranolol have been shown to reduce late-stage AMD risk by 30%, likely through improved choroidal perfusion and suppression of interleukin-6 (IL-6). In contrast, thiazide diuretics are associated with a 45% increased risk of AMD in women—an effect that appears to be mitigated when co-administered with ACE inhibitors or angiotensin receptor blockers (Xu et al., 2020; Faura et al., 2022; Luo et al., 2023). Dyslipidemia also contributes to AMD risk in a nonlinear fashion, with both very high (≥77 mg/dL) and very low (<40 mg/dL) high-density lipoprotein cholesterol (HDL-C) levels linked to increased susceptibility. Genetic polymorphisms related to lipid metabolism - such as CETP rs173539 and COLEC12 rs1999930 - further influence this relationship (Chen et al., 2025). A Korean study reported a 52% increased risk of AMD in individuals with hyperlipidemia (aHR = 1.52), while long-term statin use (e.g., atorvastatin for ≥5 years) was associated with a dose-dependent risk reduction (aHR = 0.70), likely due to statins’ anti-inflammatory and antioxidant properties (Chen et al., 2023). These findings support the potential for statins to play a dual role in cardiovascular risk reduction and AMD progression, promoting an integrated treatment strategy that addresses both systemic and ocular health.

Data from Iran showed a 6.4% prevalence of AMD among patients with hyperlipidemia, with increased risk observed in those with concurrent hypertension and diabetes, emphasizing the broader impact of metabolic syndrome on ocular health (Panahi et al., 2023). The relationship between diabetes and AMD is complex and heterogeneous. For instance, newly diagnosed diabetic patients face a 30% increased risk of wAMD, while insulin-treated individuals show a 23% higher incidence (aHR = 1.23). Those with vision-threatening diabetic retinopathy (DR) have an even greater risk (aHR = 1.35), likely mediated by hyperglycemia-induced VEGF activation (Hwang et al., 2023; Lee et al., 2023). Meta-analyses affirm a significant association between diabetes and advanced AMD (OR = 1.38, 95% CI: 1.12–1.71), although cross-sectional studies have reported inconsistent findings regarding wAMD prevalence (Zhang Y. P. et al., 2022; Virtanen et al., 2023). IL-6 has emerged as a key molecular mediator in this process, with systemic levels significantly elevated in late-stage age-related macular degeneration (AMD), including geographic atrophy and neovascular subtypes, while showing only marginal association with early AMD (Nahavandipour et al., 2020; Yadav et al., 2024). Within the local ocular microenvironment, IL-6 levels are closely correlated with VEGF-A and ICAM-1 expression, implicating activation of the STAT3 signaling pathway in neovascularization and disruption of the blood-retina barrier (Li et al., 2022). Systemic inflammatory markers also correlate strongly with disease severity. For instance, each 1 mg/L increase in CRP is associated with an 8.2 μm reduction in choroidal thickness. Elevated E-selectin levels not only predict AMD progression but also indicate heightened cardiovascular risk (Chen et al., 2021; Nashine et al., 2022), underscoring the shared pathophysiology between endothelial dysfunction, vascular compromise, and retinal degeneration.

The intricate interplay among systemic inflammation, vascular health, and retinal pathology calls for an integrated, cross-disciplinary approach that bridges cardiovascular and ocular care. Targeting systemic inflammation may be critical in halting AMD progression and mitigating its association with other systemic diseases.

### 6.2 Materials science in age-related macular degeneration

Materials science has revolutionized the treatment of AMD by addressing the limitations of conventional anti-VEGF therapies. Implantable drug depot systems now enable sustained release of ranibizumab for up to 6 months, eliminating the need for additional injections in 98% of patients and reducing intraocular drug level fluctuations by 60% compared to monthly dosing (Khanani et al., 2021; Patel et al., 2021). Nanotechnology-based delivery platforms have significantly enhanced targeting efficiency. For example, pH-sensitive PLGA nanoparticles extend the vitreous half-life of bevacizumab from 9.8 to 34.5 days, while RGD-modified liposomes achieve fivefold higher drug accumulation at lesion sites (Chen X. et al., 2024; Xu et al., 2024). Synthetic HDL nanoparticles engineered to deliver rapamycin to retinal pigment epithelial (RPE) cells have demonstrated a 68% reduction in choroidal neovascularization (Mei et al., 2022). Hydrogels, as multifunctional carriers, also show great promise. Nanofiber hydrogels co-loaded with dexamethasone and ranibizumab prolonged anti-VEGF efficacy up to 12 weeks in rabbit models and reduced vitreous inflammation by 73% (Gao et al., 2023). Gene therapy represents another major advancement. Adeno-associated virus (AAV) vectors, such as RGX-314 delivered via subretinal injection, can suppress VEGF expression for over 2 years. In phase I/IIa clinical trials, 84% of patients required no additional treatment during the study period (Campochiaro et al., 2024). This approach offers the potential to replace years of repeated intravitreal injections with a single, long-lasting intervention, exemplifying the promise of regenerative medicine for chronic ocular conditions.

In the regenerative domain, collagen glue hydrogels that mimic the biomechanical properties of the native extracellular matrix have been used to support the differentiation of human embryonic stem cells into RPE-like cells. These constructs enhanced photoreceptor survival by 41% following transplantation (Moyo et al., 2024). Interdisciplinary innovations are shifting AMD therapy from passive treatment toward precision modulation. For example, the novel rGO/PBASE electrochemical biosensor enables rapid detection of complement C3 protein within 15 min (limit of detection: 0.43 ng/mL), offering a high-sensitivity tool for early AMD screening and laying the foundation for integrated diagnostic–therapeutic systems (Ghosh et al., 2024). In parallel, artificial intelligence is being increasingly applied to clinical decision-making. Deep learning models have been shown to improve AMD staging accuracy and treatment response prediction by 23% (Crincoli et al., 2024). These models, when integrated with biosensor-derived data, can guide personalized drug administration and optimize individualized care strategies for AMD patients. Despite these promising advances, challenges remain. Issues such as long-term stability (e.g., acidic microenvironments generated by PLGA degradation) and immune compatibility (e.g., immunogenicity of PEGylated liposomes) need to be resolved (Bonechi et al., 2023; Marquina et al., 2023). Nevertheless, these innovations collectively mark a paradigm shift in AMD management - from repetitive, reactive interventions to personalized, sustained, and precision-based therapeutic strategies.

## 7 Non-arteritic anterior ischemic optic neuropathy

Non-arteritic anterior ischemic optic neuropathy (NAION) is an acute optic neuropathy strongly associated with vascular dysfunction. Its pathogenesis involves the synergistic interaction of multiple cardiovascular and metabolic risk factors.

### 7.1 Pathological mechanisms of NAION induced by cardiovascular risk factors

Systematic reviews and meta-analyses have identified hypertension, diabetes, and hyperlipidemia as independent risk factors for NAION. In particular, malignant hypertension can directly induce optic disc edema, serving as a direct trigger for NAION (Liu et al., 2021; Chatziralli et al., 2022; Miralles Pechuan et al., 2024). Notably, hypertension poses a dual threat by increasing the incidence of NAION and elevating the risk of concomitant cerebral infarction (Li X. et al., 2023). Hyperlipidemia, especially elevated triglycerides (SMD = +0.58, 95% CI: +0.12 to +1.04) and lipoprotein(a) levels (OR = 2.88, 95% CI: 1.01–8.21)—worsens optic nerve ischemia by promoting atherosclerotic changes (Chatziralli et al., 2022). The association between diabetes mellitus and an increased risk of NAION is well documented (Chen et al., 2013; Sharma et al., 2017). While visual prognosis in diabetic patients may not differ significantly from that in non-diabetics, coexisting cardiovascular conditions such as ischemic heart disease may further exacerbate optic nerve injury (Sharma et al., 2017). Among individuals with metabolic syndrome, key contributors to NAION include hyperglycemia, elevated triglycerides, and low high-density lipoprotein (HDL) levels (Kohli et al., 2022). Recent attention has focused on a potential link between glucagon-like peptide-1 receptor agonist, especially semaglutide, and NAION. This association presents a clinical paradox: while semaglutide provides substantial benefits in glucose regulation and cardiovascular protection, studies have reported a significantly increased NAION risk in patients with obesity or diabetes (hazard ratio [HR] = 4.28–7.64; cumulative incidence = 8.9%). However, broader population studies show a more modest association (HR = 1.32), highlighting the influence of population heterogeneity (Shin et al., 2021; Grauslund et al., 2024; Cai et al., 2025; Chou et al., 2025). Given these findings, clinicians should weigh semaglutide’s benefits against its potential ocular risks, particularly in high-risk patients, and prioritize optic disc evaluation and regular ophthalmic follow-up (Malerbi and Bertoluci, 2025). Furthermore, obstructive sleep apnea syndrome significantly increases the risk of NAION (RR = 3.28, 95% CI: 2.08–5.17) and coronary heart disease (RR = 1.68, 95% CI: 1.24–2.27). Inherited thrombophilic conditions, such as Factor V Leiden mutation, have also been associated with NAION (RR = 2.21, 95% CI: 1.19–4.09) (Liu et al., 2021).

### 7.2 Application of materials science in NAION

Although the application of materials science in the treatment of non-arteritic anterior ischemic optic neuropathy (NAION) remains in the exploratory phase, early advances show promising potential. Notably, curcumin-polydopamine nanocomposite hydrogels (Cur@PDA@GelCA) have demonstrated significant neuroprotective effects in optic nerve injury models. These materials act by inhibiting reactive oxygen species (ROS)-mediated oxidative stress, indicating potential utility for acute-phase intervention in NAION.

In addition to their therapeutic efficacy, the hydrogel’s strong tissue adhesion and excellent biocompatibility highlight its suitability for localized drug delivery and targeted treatment (Liu et al., 2024). These findings underscore the broader feasibility of integrating materials science into NAION therapy.

Future research should aim to further elucidate the molecular mechanisms underlying NAION and leverage multi-omics technologies alongside biomaterials innovation to develop personalized treatment strategies that can improve patient outcomes.

## 8 Outlook and summary

Materials science is increasingly recognized as a pivotal avenue for addressing ocular diseases associated with cardiovascular risk factors. Innovations such as biosensor-integrated smart contact lenses, PLGA-based nanocarriers, and neuroprotective hydrogels are driving therapeutic strategies toward greater precision, reduced invasiveness, and prolonged efficacy (Table 1). However, several critical challenges remain, such as the acidic microenvironment generated by PLGA degradation, the long-term biocompatibility of implants, and the limited efficiency of clinical translation.

**TABLE 1:** Materials for cardiovascular risk-linked ocular diseases.

Material type	Application	Target disease(s)	Advantages
Smart contact lenses	Real-time monitoring of intraocular pressure, tear glucose, or inflammatory markers	DED (Yetisen et al., 2020; Mondal et al., 2023), Glaucoma (Kim et al., 2022; Zhu et al., 2022; Kompella et al., 2021)	Non-invasive, continuous, patient-friendly monitoring
PLGA nanoparticles	Sustained release of anti-VEGF, corticosteroids, or neuroprotectants	Glaucoma (Mahaling and Katti, 2016), DR (Rong et al., 2019), AMD (Chen et al., 2024b; Xu et al., 2024; Bonechi et al., 2023; Marquina et al., 2023)	Long-acting, high drug-loading capacity, biodegradable
Liposomes/exosomes	Encapsulation and targeted delivery of mRNA, siRNA, or protein drugs	DR (Wang et al., 2024b), AMD (Chen et al., 2024b; Xu et al., 2024), Glaucoma (Liu et al., 2020a; Durmaz et al., 2024), Hyperuricemia-Related Ocular Diseases (Ma et al., 2025)	Biocompatible, precise targeting, reduced systemic exposure
Hydrogel-based drug systems	Controlled release of anti-inflammatory or anti-scarring agents post-surgery	Glaucoma (post-surgery) (Lin et al., 2023), NAION (Liu et al., 2024)	Reduces surgical complications, sustained anti-scarring effect
Microneedle patches	Transscleral or subconjunctival delivery of uric acid-lowering drugs	Hyperuricemia-associated inflammation (Patel and Thakkar, 2023)	Minimally invasive, improves drug bioavailability
Peptide supramolecular systems	Sequestration of inflammatory cytokines, immune modulation	DR (Li et al., 2023b), AMD (Gao et al., 2023)	High specificity, endogenous response mimicking
Nanofiber hydrogels	Dual drug release platforms for anti-angiogenesis and anti-inflammation	AMD (Gao et al., 2023)	Multimodal therapy, controlled kinetics
LSPR biosensors	Non-invasive biomarker detection in tear or aqueous humor samples	DED (Culver et al., 2018; Wechsler et al., 2021)	High sensitivity, real-time diagnostics
Zwitterionic coatings	Surface modification to enhance immune evasion and reduce protein adsorption	All implantable materials (cross-cutting)	Improved biocompatibility, reduced immune rejection

Future research should advance along multiple fronts. In material design, pH-buffering coatings and environment-responsive modifications may help reduce local tissue irritation. For preclinical validation, organoid models and microfluidic “eye-on-a-chip” systems offer physiologically relevant platforms for evaluating safety and efficacy. To improve immune compatibility, approaches such as PEGylation and zwitterionic surface engineering can minimize inflammation and extend *in vivo* functionality.

Facilitating clinical translation will require early and proactive engagement with regulatory agencies to navigate approval pathways for nanomedicines and combination therapeutic devices. Additionally, stronger collaboration between academia and industry, paired with standardized, scalable manufacturing processes, will be essential for bridging the gap between laboratory innovation and clinical application.

In conclusion, the integration of advanced materials, biosensing technologies, and intelligent drug delivery systems offers a comprehensive and promising framework for the personalized management of ocular diseases in patients with cardiovascular comorbidities. A deeper alignment between engineering innovation and clinical practice will be vital to achieving long-lasting, widely applicable therapeutic outcomes.

## Abbreviations

AGE, advanced glycation end; AMD, age-related macular degeneration; hs-CR, Phigh-sensitivity C-reactive protein; DED, dry eye disease; DR, diabetic retinopathy; ERS, endoplasmic reticulum stress; HDL, high-density lipoprotein; HTN, hypertension; IL-6, interleukin-6; IOP, intraocular pressure; LSPR, localized surface plasmon resonance; MAGNs, Manganese-doped albumin nanogels; MGD, meibomian gland dysfunction; MMC, mitomycin C; MMP-9, metalloproteinase-9; NAION, non-arteritic anterior ischemic optic neuropathy; OAG, open-angle glaucoma; POAG, primary open-angle glaucoma; RGC, retinal ganglion cell; RGD, arginine-glycine-aspartate; RNFL, retinal nerve fiber layer; ROS, reactive oxygen species; RPE, retinal pigment epithelium; UA, uric acid; wAMD, wet age-related macular degeneration.
